# Blunt Trauma to the Common Femoral Artery From Motorcycle Handlebar Impact: A Case of Extensive Thrombosis

**DOI:** 10.7759/cureus.86291

**Published:** 2025-06-18

**Authors:** Santhosh Balachandra, Madhur Uniyal, Amit Pareek, Rohit Chauhan, Ayush Jaiswal

**Affiliations:** 1 Trauma Surgery and Critical Care, All India Institute of Medical Sciences, Rishikesh, Rishikesh, IND; 2 Trauma and Acute Care Surgery, All India Institute of Medical Sciences, Rishikesh, Rishikesh, IND; 3 Trauma and Critical Care Surgery, All India Institute of Medical Sciences, Rishikesh, Rishikesh, IND

**Keywords:** blunt vascular injury, common femoral artery, handlebar trauma, lower limb ischemia, thrombectomy

## Abstract

Blunt trauma can cause significant vascular injury even in the absence of fractures or penetrating wounds, and such injuries are often underdiagnosed. In this case, the rarity lies in the intraoperative identification of an unusually extensive thrombus measuring 71 cm in length - an exceptional finding rarely described in blunt vascular trauma. We report a case of a 59-year-old male who sustained trauma to the left groin from a motorcycle handlebar impact during a road traffic accident. He presented six hours after the incident with absent distal pulses and delayed capillary refill in the affected limb. Computed tomography angiography revealed complete non-opacification of the left common femoral artery and proximal superficial femoral artery over a 4.4 cm segment, suggestive of arterial thrombosis. Emergency surgical exploration confirmed the thrombus, which was successfully extracted using a Fogarty catheter. The contused arterial segment was resected, and a tension-free end-to-end anastomosis was completed using a 6-0 Prolene suture. Based on clinical assessment, the limb ischemia was classified as Rutherford Grade IIa, indicating a marginally threatened limb requiring prompt intervention. Postoperatively, limb perfusion was restored with no signs of ischemic complications, and the patient regained full function. At six-week follow-up, triphasic flow was confirmed on Doppler ultrasound. This case underscores the importance of maintaining a high index of suspicion for vascular injury in blunt trauma patients, particularly when skeletal injury is absent. Early diagnosis and timely surgical management are vital to prevent limb loss and ensure favorable outcomes.

## Introduction

Vascular trauma, though uncommon, is a significant cause of morbidity and mortality in trauma patients. Globally, it accounts for less than 3% of all reported injuries but poses a high risk of limb loss and functional impairment if not promptly identified and managed [1,2]. In India, although comprehensive national data are limited, a few institutional studies have reported vascular injury incidence ranging from 1.5% to 2.3% among trauma admissions at tertiary centers [3]. While penetrating trauma is the predominant mechanism in most vascular injuries, blunt vascular trauma remains underrecognized, particularly in cases lacking concomitant skeletal damage or overt signs such as pulsatile bleeding [4].

Blunt vascular injuries may result from shear stress, compression, or rapid deceleration forces, leading to arterial intimal damage, thrombosis, dissection, or even transection. Among peripheral arteries, the common femoral artery (CFA) is relatively well protected by its anatomical location beneath the inguinal ligament and adjacent muscular structures. However, blunt trauma to the groin - such as that from motorcycle handlebars - can still cause significant vascular disruption even in the absence of bony injury [2].

CFA injuries constitute approximately 3%-6% of all peripheral arterial injuries, and blunt trauma accounts for only 5%-10% of CFA trauma cases, making such presentations particularly rare [5,6]. Motorcycle handlebar injuries are an unusual mechanism, most often reported in pediatric abdominal trauma and only sporadically documented as a cause of isolated CFA thrombosis in adults [7].

These injuries may present with subtle clinical findings, including localized swelling, ecchymosis, or diminished distal pulses. The absence of “hard signs” of vascular injury - such as active hemorrhage, expanding hematoma, bruit, or thrill - can delay diagnosis and appropriate intervention [8]. In the setting of blunt trauma, differential diagnoses for limb ischemia include arterial thrombosis, intimal flap or dissection, traumatic arterial spasm, compartment syndrome, and embolism from a proximal injury site. Early imaging, especially with duplex Doppler ultrasound and computed tomography angiography (CTA), plays a pivotal role in identifying vascular occlusions, pseudoaneurysms, or flow deficits [9,10].

Timely surgical exploration is critical to re-establish limb perfusion and prevent complications such as ischemia-reperfusion injury, muscle necrosis, compartment syndrome, and, ultimately, limb loss [11]. The importance of considering vascular injury in any blunt trauma patient with clinical features of ischemia - even without radiologic evidence of fracture - cannot be overstated. In this report, we present a unique case of blunt trauma-induced CFA thrombosis following motorcycle handlebar impact, which resulted in the retrieval of an unusually long thrombus.

## Case presentation

**Figure 1:**
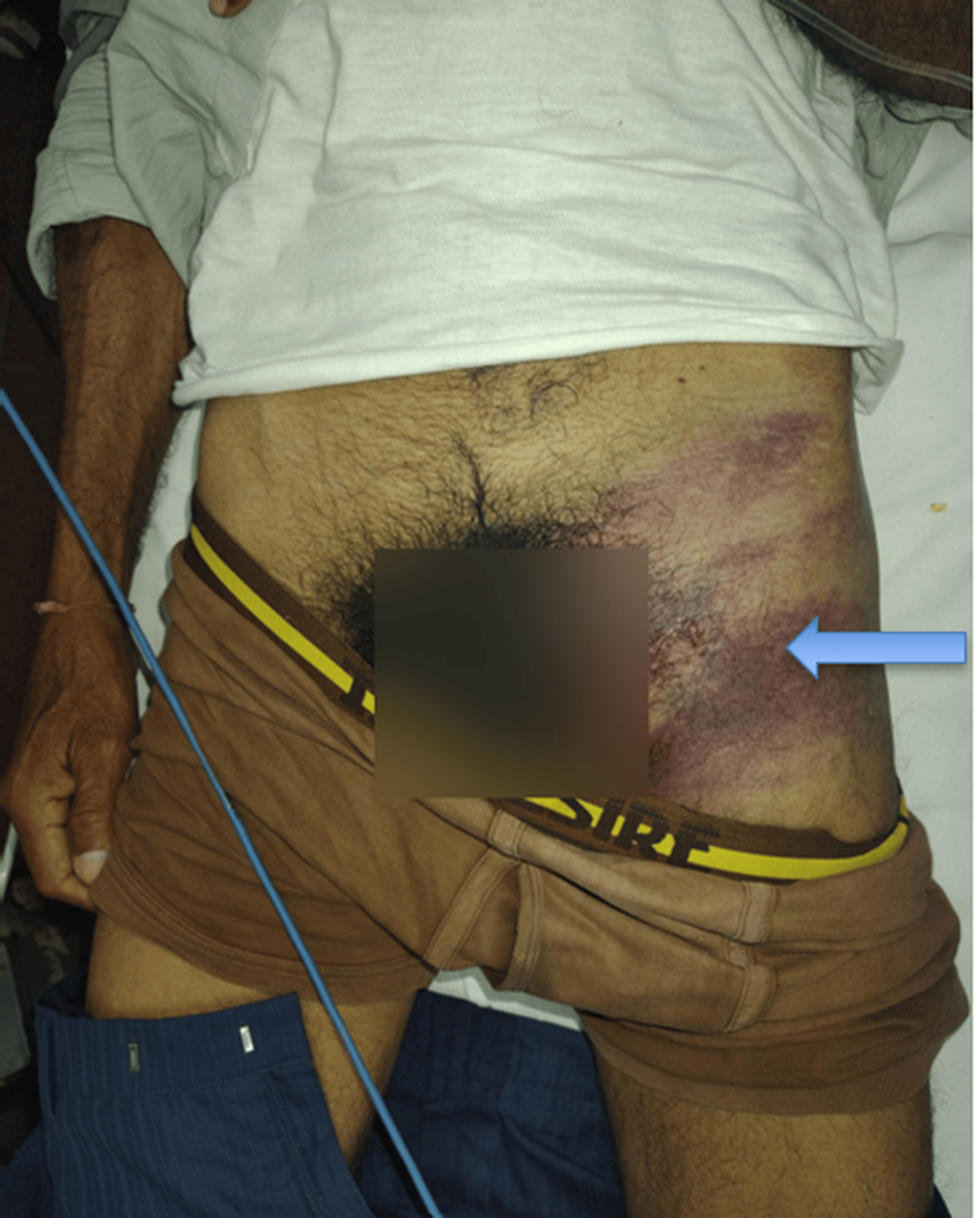
Clinical photograph of the left groin showing the ecchymotic patch Ecchymosis over the left proximal thigh and inguinal region following blunt trauma from a motorcycle handlebar. The blue arrow indicates the area of subcutaneous discoloration.

A 59-year-old male presented to our level 1 trauma center six hours after a road traffic accident involving a collision with an auto-rickshaw. He sustained blunt trauma to the left groin from the motorcycle handlebar. On arrival, he was hemodynamically stable. The primary survey was unremarkable with no evidence of head, chest, or abdominal injury. On secondary survey, local examination of the left groin revealed a 10 × 10 cm ecchymotic patch with localized tenderness but no external wounds, deformity, or palpable hematoma (Figure 1).

Distal pulses - including the popliteal, posterior tibial, anterior tibial, and dorsalis pedis arteries - were absent in the left lower limb. Capillary refill time was delayed, SpO₂ was undetectable in the toes, and bedside Doppler confirmed the absence of arterial flow beyond the femoral segment. The left limb was soft, non-tense, and not tender on passive stretch. Sensory and motor examinations were grossly intact, and no signs of compartment syndrome were present. Due to clinical signs of acute limb ischemia, an urgent CT angiogram of the lower limb was performed. Imaging revealed complete non-opacification of the left common femoral artery and proximal superficial femoral artery over a 4.4 cm segment. Additionally, there was absent distal flow in both the anterior and posterior tibial arteries, consistent with thrombotic occlusion.

**Figure 2:**
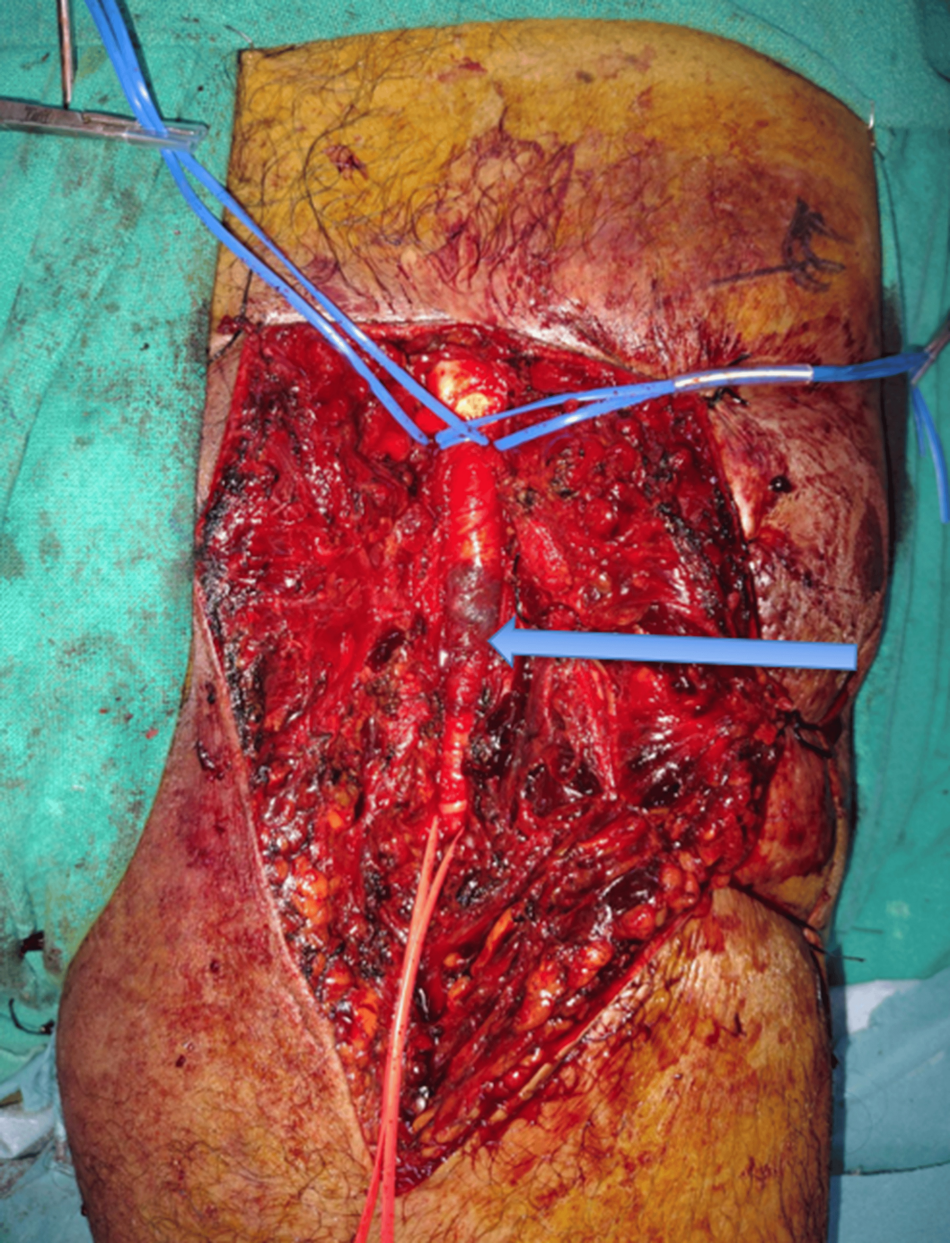
Intraoperative image showing common femoral artery injury Intraoperative image showing a contused and thrombosed segment of the left common femoral artery. The blue arrow indicates the injured arterial segment. Vessel loops are placed for proximal and distal control.

After obtaining informed consent for emergency intervention with possible limb salvage or amputation, the patient was shifted to the operating room. The contralateral great saphenous vein was marked and prepared in anticipation of a potential interposition graft. Through a longitudinal groin incision, surgical dissection revealed a 4 cm contused and thrombosed segment of the common femoral artery with surrounding hematoma and absent distal pulsations (Figure 2).

**Figure 3:**
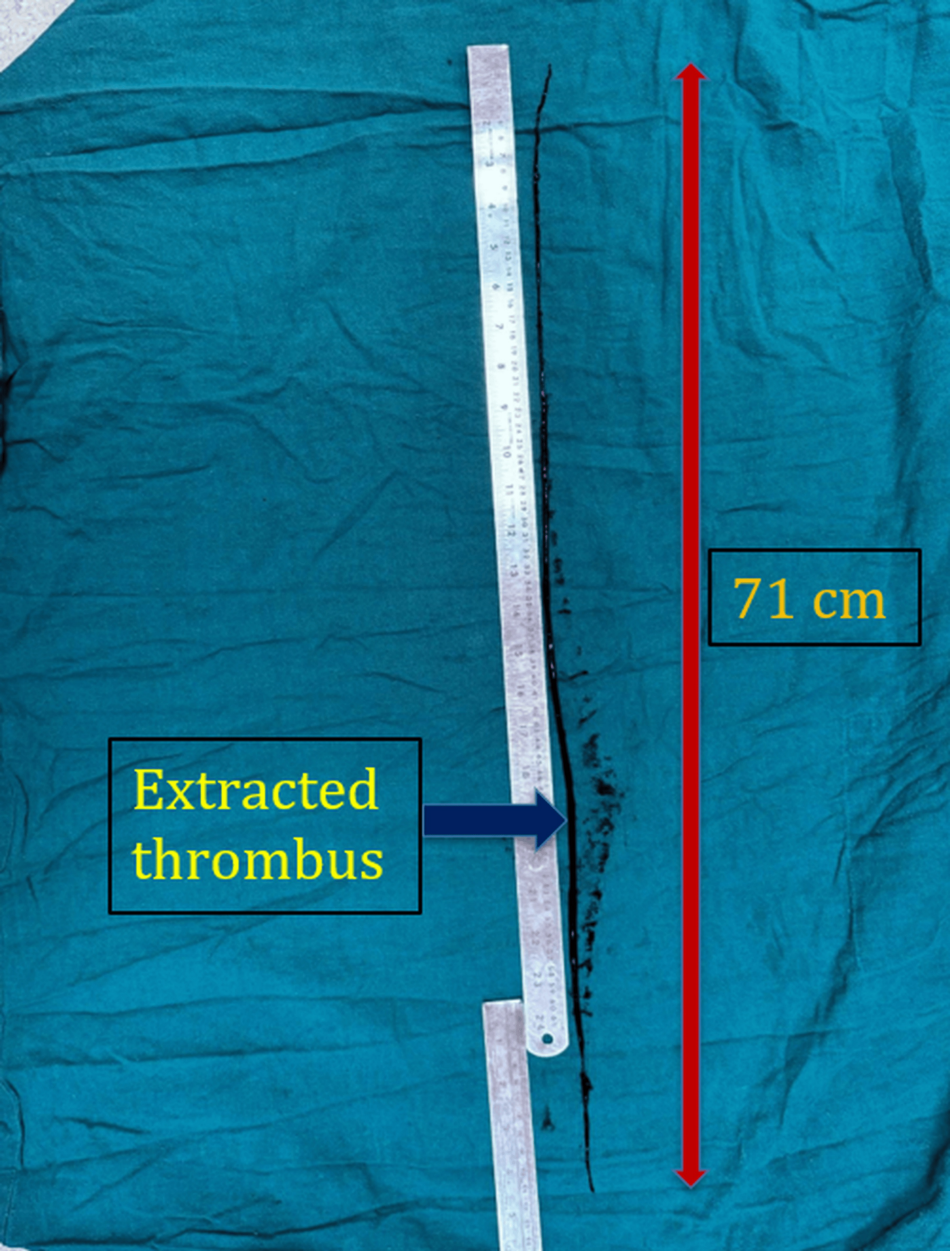
Extracted thrombus following femoral artery thrombectomy A 71 cm thrombus extracted via a Fogarty catheter from the common femoral and superficial femoral arteries, displayed alongside a measuring scale. The red arrow indicates the total measured length; the blue arrow indicates the extracted thrombus.

Intraoperatively, a thrombus measuring 71 cm in length was extracted in a single segment using a Fogarty catheter. The thrombus extended proximally from the CFA origin and involved the entire superficial femoral artery (SFA) up to its distal segment, without reaching the popliteal artery. No proximal aortic or iliac source of embolism was identified, supporting an in-situ thrombotic mechanism secondary to blunt endothelial injury at the CFA (Figure 3).

**Figure 4:**
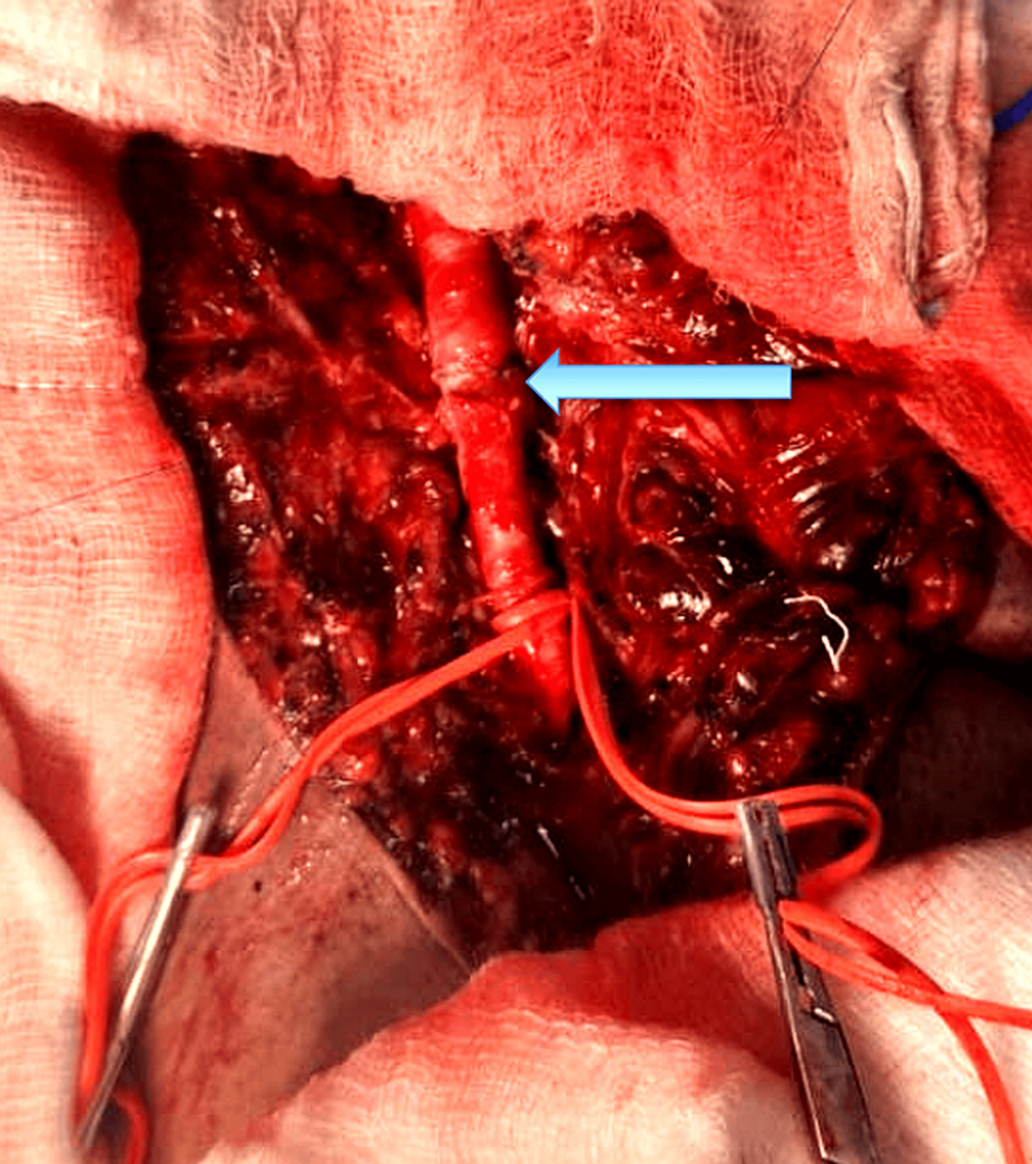
Intraoperative image showing completed end-to-end arterial anastomosis Tension-free end-to-end anastomosis of the left common femoral artery following excision of the contused segment. The blue arrow indicates the arterial repair site. Vessel loops are in place for intraoperative vascular control.

The thrombosed and damaged arterial segment was excised. Given the adequacy of the remaining arterial length, a primary end-to-end anastomosis was completed using 6-0 Prolene suture under loupe magnification, ensuring a tension-free repair (Figure 4). 

An intraoperative waiting period was observed to monitor for reperfusion syndrome and to confirm distal flow restoration. Following anastomosis, distal arterial pulsations were promptly restored, capillary refill normalized, and SpO_2_ in the left toes improved to 100%. The limb was wrapped in a sterile dressing and immobilized in a flexed position to prevent kinking or tension on the repair site. Postoperative monitoring focused on early detection of re-thrombosis, ischemia-reperfusion injury, and compartment syndrome. No such complications were observed.

The patient was initiated on aspirin 75 mg daily and underwent passive range-of-motion physiotherapy beginning on postoperative day one. He remained stable throughout his hospital course and was discharged on day five with full limb perfusion and intact neurologic function. At six-week follow-up, he was ambulating independently with full weight-bearing capacity and no evidence of muscle atrophy. A Doppler ultrasound examination demonstrated triphasic flow in the femoral, popliteal, and tibial arteries, confirming the long-term patency of the repair.

## Discussion

Vascular injuries are a critical component of trauma care due to their potential for rapid deterioration and limb-threatening consequences. Although penetrating trauma is the leading cause, blunt trauma must not be underestimated, especially when high-velocity impacts or localized compression forces are involved [4]. Blunt vascular injuries can result from motor vehicle collisions, falls, or direct blows, such as handlebar trauma, and are associated with high rates of delayed diagnosis due to a lack of overt skeletal damage or external bleeding [9].

The CFA lies deep to the inguinal ligament and is shielded by surrounding musculature, making injuries rare in the absence of associated skeletal trauma. However, focused compression, particularly in motorcycle accidents, can result in localized intimal injury, thrombus formation, and eventual occlusion [12].

The key to successful outcomes in vascular trauma lies in early diagnosis and prompt intervention. Delayed recognition can result in irreversible ischemic damage, rhabdomyolysis, multiorgan failure, or amputation [13]. The “6 P’s” of limb ischemia - pain, pallor, pulselessness, paresthesia, paralysis, and poikilothermia - should be actively sought. In the absence of clear signs, bedside Doppler and CTA serve as non-invasive, high-yield diagnostic tools [7,14].

Previous reports, such as those by Neville et al. [15] and Clouse et al. [16], have documented thrombus lengths ranging between 25-50 cm in trauma-related arterial occlusions. However, our case demonstrates an exceptionally long thrombus (71 cm), surpassing prior descriptions and emphasizing the extent of thrombosis that can occur from localized intimal damage without associated fracture or dissection. Unlike these earlier cases, where thrombus extension was often associated with proximal embolic sources or delayed presentation, our patient developed this extensive occlusion within hours of trauma, suggesting a hyperacute in-situ propagation.

The discrepancy between the 4.4 cm segmental non-opacification seen on CTA and the 71 cm thrombus retrieved intraoperatively can be attributed to limitations in CT resolution and flow dynamics distal to a complete occlusion. The thrombus likely propagated distally within a low-flow or stasis environment created by the abrupt endothelial injury at the CFA. In-situ thrombosis extending along the superficial femoral artery may have occurred silently, without contrast filling, and was only fully appreciated during surgical retrieval.

While open surgical thrombectomy and primary repair were feasible and effective in our case, alternative strategies such as catheter-directed thrombolysis, percutaneous mechanical thrombectomy, or stent-grafting may be considered in patients who are poor surgical candidates or present with more distal occlusions. However, such endovascular interventions require cautious patient selection, particularly in the context of trauma-induced vascular injuries, where vessel wall integrity may be compromised.

Surgical management typically involves proximal and distal vascular control, thrombectomy using a Fogarty catheter, and repair via end-to-end anastomosis or interposition graft, depending on the extent of arterial damage [17]. In our case, despite the length of the thrombosis, a primary end-to-end anastomosis was possible after resecting the contused arterial segment. Use of fine Prolene suture and meticulous technique ensured a tension-free repair.

Postoperative management should include vigilant monitoring for reperfusion syndrome, compartment syndrome, and re-thrombosis. Antiplatelet therapy with aspirin and limb elevation is are standard adjunct. Our patient recovered fully, underscoring that even extensive injuries, if diagnosed and treated early, can lead to excellent functional outcomes [18].

## Conclusions

This case highlights the critical need for heightened clinical vigilance in patients presenting with blunt trauma to anatomically sensitive regions such as the groin, even in the absence of overt skeletal injury or penetrating wounds. Delayed recognition of vascular injury can lead to devastating consequences, including irreversible limb ischemia and potential limb loss. Timely vascular imaging, meticulous surgical technique, and structured postoperative monitoring are essential for successful limb salvage. Notably, the retrieval of a 71 cm thrombus from the femoral arterial system represents an exceptionally rare finding in blunt vascular trauma, underscoring the severity such injuries can reach even in the absence of fractures. Our experience demonstrates that even low-velocity blunt trauma can result in extensive arterial thrombosis and that early intervention can lead to complete functional recovery. To improve outcomes, we recommend incorporating formal vascular assessments - including serial pulse checks and early use of duplex ultrasonography or CTA - into initial trauma evaluation protocols for patients with localized extremity trauma. Greater clinical awareness and structured diagnostic algorithms can enhance early detection and timely management of these rare but potentially limb-threatening vascular injuries.
